# Viewing Mobile Health Technology Design Through the Lens of Amplification Theory

**DOI:** 10.2196/31069

**Published:** 2022-06-10

**Authors:** Beza Merid, Maria Cielito Robles, Brahmajee K Nallamothu, Mark W Newman, Lesli E Skolarus

**Affiliations:** 1 School for the Future of Innovation in Society Arizona State University Tempe, AZ United States; 2 Department of Neurology University of Michigan Medical School Ann Arbor, MI United States; 3 Department of Internal Medicine University of Michigan Medical School Ann Arbor, MI United States; 4 School of Information University of Michigan Ann Arbor, MI United States

**Keywords:** mHealth, digital health, cardiovascular disease, high blood pressure, structural barriers to health, racial health disparities, Amplification Theory of Technology

## Abstract

Digital health interventions designed to promote health equity can be valuable tools in the delivery of health care to hardly served patient populations. But if the design of these technologies and the interventions in which they are deployed do not address the myriad structural barriers to care that minoritized patients, patients in rural areas, and patients who have trouble paying for care often face, their impact may be limited. Drawing on our mobile health (mHealth) research in the arena of cardiovascular care and blood pressure management, this viewpoint argues that health care providers and researchers should tend to structural barriers to care as a part of their digital health intervention design. Our 3-step predesign framework, informed by the Amplification Theory of Technology, offers a model that interventionists can follow to address these concerns.

## Introduction

Heart disease is a leading cause of death in the United States, killing roughly 655,000 Americans each year [1]. It also represents a disproportionate harm to minoritized people, who often face structural barriers to health including poor access to emergency medical services and treatment, insurance coverage, healthy foods, and safe environments for physical activity [2,3]. Efforts to monitor and prevent heart disease focus on the prevalence of key risk factors—including uncontrolled blood pressure and low-density lipoprotein cholesterol, a history of smoking, physical inactivity, and poor diet—and the role that health care providers and patients themselves can play in eliminating or minimizing their effect on patient and population health [4]. These risk factors in particular are prominent opportunities for intervention because, unlike other sources of risk such as age and family history, they are considered “modifiable” and, thus, an opportunity for providers to prevent disease and for patients to take action to secure their own health.

The relationship between providers and patients here revolves around the implementation of disease prevention strategies that are both effective in reducing morbidity and mortality while also being achievable within the resource constraints that shape health care delivery and patients’ daily lives. These strategies, in other words, try to offer practical solutions to address health care needs using tools, technologies, and means of communication that should be widely available to the populations they seek to serve. For example, researchers and providers studying racial health disparities in cardiovascular disease treatment and outcomes use SMS text messaging to facilitate communication between providers and patients; electronic home blood pressure monitors to enable the tracking of trends in blood pressure readings over time; and wearable devices such as the Fitbit and Apple Watch to monitor health metrics such as heart rate, exercise, and cardiac electrical activity [5,6]. These are used because they rely on technologies that are both accessible to patients on the consumer technology market and, with regard to the use of SMS text messages and activity trackers on smartphones, they make use of functions that are native to these devices and easy for users to incorporate in their daily lives.

While this approach to using digital health technologies to address modifiable risk factors for disease is an important modality of care, this viewpoint argues that *access* to these technologies does not guarantee the ability to afford or sustainably use them; it is merely one precondition of technology use that providers and researchers should consider when designing technological interventions to address patient needs. Equitably designed digital health interventions must also account for structural determinants of health that may shape how patients of different races, ages, and socioeconomic status, among other characteristics, would fare when encountering these interventions. This paper provides a predesign framework that interventionist health services researchers can implement, *prior to* deploying their digital health interventions, to think about facilitators of and barriers to technology use among patients whose resource constraints may shape their capability, opportunity, or motivation to address modifiable cardiovascular disease risk factors. We conclude by providing a case study where we apply this model in our ongoing work in this space.

## Techno-Optimist Versus Techno-Pessimist Views of Mobile Health Interventions

Despite what the ubiquity of technologies such as smartphones and wearable devices might suggest about the promise and value of new technologies in our ongoing efforts to limit the disproportionate harm of cardiovascular disease on minoritized communities, their widespread commercial availability belies a fundamental tension about what we believe technology can do to address such disparities. This tension, broadly speaking, is between what we can call techno-optimist and techno-pessimist views of technology [7]. The former tends to view technology itself as additive or transformative, presuming that access to a given device is enough to create a desired change within the lives of its imagined users; the latter tends to believe that, without the provision of supportive infrastructures and attention to users’ specific needs and barriers to use, technology itself may simply amplify existing inequities in access or opportunity. This acknowledgement of the need to think reflexively about technology and what we believe it can do is a central tenet of the “Amplification Theory of Technology,” which calls on interventionist researchers to account for how the social conditions in which technologies are deployed fundamentally shape how—and if—they can be used.

In the context of health broadly, and mobile health (mHealth) interventions in particular, this theory explains how technologies can amplify adverse social determinants of health if they are not designed and deployed in a manner that is congruent with users’ capabilities to use them. Many researchers and providers tend to align themselves toward the techno-pessimist position, worrying, for example, about a widening digital divide and the risk of creating “intervention-generated inequalities” [8]. This is an important concern, and it should be used to inform digital health technology and intervention design. The model for mHealth research that we propose here adopts this theory, and it asserts that techno-pessimism and a continued effort to develop technologies that address patients’ needs are not only compatible positions for us to hold, but also part of a requisite relationship with technology itself.

## Amplification Theory of Technology

We argue that this Amplification Theory of Technology, as formulated by Kentaro Toyama [7], should inform our efforts in designing mHealth interventions and, critically, the work we can do to ensure the safe and sustained use of these technologies by patients in underserved communities. The Amplification Theory described in Toyama’s work makes 3 assertions.

First, it argues that technology cannot function as a substitute for institutional capacities or human intent that is missing among stakeholders or environments where an intervention is to be deployed; this is because technology is not a fixed force that, on its own, causes certain kinds of social change [7]. Such interventions require a scaffolding of social, political, and technological infrastructures to support the equitable deployment of a given technology.

Second, the theory argues that technology tends to amplify existing inequalities. Simply making a technology *accessible* to underserved populations will not ensure that the technology is usable among these populations, and will certainly not address structural conditions such as political and social marginalization, or a differential distribution of lifesaving resources. In contrast to a theory of technology that presupposes either a positive or a negative directionality of effect as a fixed impact, the Amplification Theory of Technology argues that technology is merely a tool “that multiplies human capacity in the direction of human intent” [7].

Third, the theory argues that technologies are most effective when they amplify successful intervention efforts with existing institutional capacity and intent to foster positive change, rather than attempting to fix or substitute for “missing institutional elements” [7]. Technologies can have both positive and negative effects, because they are magnifiers of human intent and capacity. This framing is in contrast to a view that might posit that universal access to a technology would function as a silver bullet for social problems.

We are interested in this kind of direct investment in human capacity and opportunity to use technology as part of our community-engaged research in Flint and Ann Arbor, Michigan, where we are working with community members with hypertension to develop an mHealth intervention that promotes physical activity and nutrition to help control blood pressure and prevent heart disease and stroke. We illustrate here how the development of mHealth interventions through the lens of Amplification Theory can help to negotiate the tension between techno-optimist and techno-pessimist positions and, further, provide a road map that health care providers and researchers can use to design interventions that use technology as support and an amplification of a broader social intervention to address persistent health disparities.

## mHealth Technologies: Promises and Limitations

For health care providers and researchers who address disease disparities across the diagnostic spectrum, mHealth interventions may offer a sense of great promise in their capacity to deliver and improve health care. The use of SMS text messaging and smartphone apps to educate patients, “nudge” behavior change [9], enable continuous health monitoring [10], provide access to patient health information, and facilitate patient-provider communication [11] can generate impactful new ways to support patients and promote health. Building on existing efforts by patients to involve themselves in the management of their care and in decision-making processes, these interventions can enable patients to become “digitally engaged” [12] by adopting new media technologies that facilitate self-management. These types of interventions are informed by surveys and scholarship indicating that hardly reached populations—including minoritized people, people in rural areas, and people who may otherwise have trouble paying for health care—typically already have a mobile device such as a smartphone that can be used to this end [5,11]. The possibility of reaching these patients who may already have the capability and opportunity to use these technologies is exciting.

Attention to high rates of utilization of mobile devices among these populations is often a central focus of studies advocating for and evaluating the use of mHealth interventions, particularly among minoritized people [13]. Some researchers argue that, in the midst of a growing digital divide that exacerbates the harms of racism in health care, low-wage employment, and poor access to hospital facilities and providers, use and ownership of mobile devices can create a new means to self-manage disease risk and illness. They also suggest that mHealth interventions can offer a sense of social support to users by underscoring the value of health-promoting behaviors [14], thereby offering patients agency and a social infrastructure through which they can manage their health risks and outcomes.

However, these high rates of smartphone utilization do not tell the whole story. For example, in addition to documenting the near ubiquity of smartphone ownership, Pew Research reports that a plurality of smartphone owners say they use their smartphones, rather than a computer, to go online. But these data also indicate that there are notable demographic differences in this usage, including important distinctions by age. Pew notes that 60% of smartphone users between the ages of 18 and 29 and 51% of smartphone users aged 30-49 prefer using mobile devices to go online, as compared with 34% of users aged 50-64 and 28% of users aged 65 and older; conversely, 42% of smartphone users aged 50-64 and 44% of smartphone users aged 65 and older prefer using desktops, laptops, or tablets to access the internet, as compared with 22% of users aged 18-29 and 21% of users aged 30-49 [13]. These findings illustrate how the big picture of smartphone usage changes when we look at it with some granularity, in this case by comparing population segments by age. They also illustrate how, for example, an mHealth intervention that seeks to prioritize older adults would need to think carefully about the digital health strategies being employed, as well as the preferences and capabilities of the patients they hope to help, lest they exacerbate existing inequities in capability, opportunity, or motivation to use these devices [15].

We are taking a similar context-sensitive approach in our work with patients with hypertension in Flint and Ann Arbor to look beyond *access* to technology to consider the social, political, and economic conditions that may facilitate or prevent the use of our mHealth intervention. One problem with focusing this kind of work on access to technology is that it can situate the underutilization of digital technologies among particular populations as a problem for the patient, rather than as a problem for a health care system that disadvantages myriad patients within particular populations or social demographics. As Veinot et al [8] argue, this focus on individuals and individual-level health behaviors can be useful in triaging patients’ emergent needs, but it misses an opportunity to work toward broader, structural solutions.

To move beyond framing this work around individual-level behavior change in digital health technology use, we deploy the COM-B (Capability, Opportunity, Motivation, Behavior) theory of behavior change to explain how structural conditions may impede patients’ use of digital health technologies to prevent cardiovascular disease [16]. As Michie et al [16] explain, the COM-B model offers a theory of behavior change that accounts for social and community networks as well as general socioeconomic, cultural, and environmental conditions that can shape a person’s capability, opportunity, or motivation to change a behavior. The focus of this model is on understanding behaviors—such as nonuse of a digital health technology recommended by a health care provider, for example—in its proper context, where the behavior can be situated as part of a broader social system. The 3 conditions necessary for behavior change, as explained by this model, are capability, opportunity, and motivation; in order to design digital health interventions that are likely to be successful, we argue, providers and researchers must think reflexively about how these conditions of behavior change may shape patients’ relationships with the technologies we deploy. We discuss our application of this theory within our ongoing work on the Wearables in Reducing risk and Enhancing Daily Lifestyle (WIRED-L) study in the section that follows.

## WIRED-L Study: Case Study

In our work at the WIRED-L Center, we engage in community-based participatory research to design an mHealth intervention that will assist patients with hypertension in lowering their blood pressure through increased physical activity and a healthy diet. Our approach to this work is informed both by this literature and our efforts to work with patient communities to understand what they would value in an mHealth intervention. Our framework for this intervention also includes a 3-step process, taking place prior to the deployment of our smartphone app, during which we apply the Amplification Theory of Technology to identify structural barriers to the use of our technological intervention as well as possible actions our collective research team can take to address these barriers and help facilitate the sustainable use of our mHealth intervention; we summarize these early stages of our community-engaged work below, and, following those details, share our 3-step process for thinking reflexively about technology in society in Table 1.

A primary goal of ours is to ensure that we are designing an intervention with—and not for—our community partners. As such, we are working with community leaders in community-based organizations focused on the health of older adults, community members who have participated in prior health studies in University of Michigan hospitals, and community members affiliated with the Community Ethics Review Board (CERB) [17] in Flint, Michigan, to discuss our shared vision for this work. The CERB is particularly important as it includes a group of community volunteers and leaders who conduct a review process to ensure that proposed research meets community needs, and that projects are sensitive to community culture.

**Table 1:** The 3-step process applying the Amplification Theory in addressing structural barriers to health technology use.

Steps	Sample questions	Examples of action
Step 1: Acknowledge the possibility of technology amplifying existing inequalities rather than transforming and immediately improving patient health	Presuming access to a given technology, what do we know about users’ capability or opportunity to use the technology at the center of our intervention? Does any institutional capability to support this intervention already exist?	Create a matrix documenting differential access or capability that may limit community partners’ use of technological intervention. Determine whether or not intervention relies on “myth of scale.”
Step 2: Name structural, environmental, and social barriers that may prevent use within specific communities and among specific users	Is the mHealth^a^ intervention we are deploying accessible, affordable, and safe to use within our partner community? What specific conditions may limit accessibility, affordability, and safety for users in this community? What are the health effects of policy decisions such as “digital redlining,” where internet service providers systematically exclude low-income neighborhoods from broadband access?	Ask participants to identify environmental barriers to safe use of mHealth interventions (eg, lack of sidewalks and public park space as a barrier to physical activity interventions). Identify existing limitations to local broadband internet connectivity, and articulate how structural barriers to information access can affect health.
Step 3: Identify and pursue coalitions to enact social, economic, and policy infrastructures needed to sustainably deploy interventions as designed	Which providers, researchers, organizations, experts, and policymakers can help answer these questions? How are we ensuring that community partners are active in this process, driving our inquiries and discussions about possible solutions? What kind of funding is necessary to sustain the benefits derived from this intervention, and what can we do to secure it?	Contact state legislature to call for allocation of public funding of broadband internet access for low-income patients and families who may benefit from mHealth intervention.

^a^mHealth: mobile health.

We interviewed community members with hypertension in both Flint and Ann Arbor to understand their capabilities, opportunities, and motivations to engage in cardiovascular disease risk factor reductions, to assess their use of technology in their daily lives, and their interest in a technological intervention to promote cardiovascular health. We also engaged in preliminary design workshops involving members of our team of health researchers and providers as well as our community partners. Our predesign research also included presentation storyboards shared with community members that created low-fidelity renderings of possible features that could be built into the mHealth app to assess and design toward our community partners’ needs.

As we engage this mHealth design process that centers the needs of our patients, we have gained several insights. For instance, our Flint participants—who are predominantly Black and who reside in a majority Black city—report a lack of safe and accessible outdoor environments that facilitate physical activity for older adults; this is a finding borne out in research on interventions that seek to deploy technologies within hardly served populations, so while it does not represent a novel discovery in this context, we include this reflection here to note that this barrier to physical activity is not experienced by our Ann Arbor participants—who are predominantly White and who reside in a majority White city—and to highlight the importance of using such disparities to inform an analysis of the political economy of health *as a part of* mHealth research. This acknowledgement of constraints in users’ capability and opportunity to use a given technology, we argue, should directly inform the design choices we make before we deploy an intervention, and the community work we engage in after deployment to sustain the use of an intervention.

To that end, we present a 3-step process (Table 1), to be carried out prior to the deployment of a technological intervention, that providers and researchers can follow to ensure that the technologies they are designing do not inadvertently exacerbate existing inequalities, and to generate ideas about how they can also address the structural conditions that sustain these inequalities in the first place.

The first step in our predesign framework calls on providers and researchers to acknowledge the possibility that the technological interventions we design may amplify existing inequalities in rather that than transform or immediately improve patient health. We are drawing here on Toyama’s [7] work on the Amplification Theory of Technology. Toyama [7] warns that we must look for the ways in which technology amplifies underlying human forces and social conditions. Asking questions about the assumptions we are making about technology, the directionality of influence we presume our technologies will have, and the differentials in access and motivation in user populations can enable us to address more directly these issues in our design, refinement, and deployment processes. The deliverable produced here should systematically document these beliefs and assumptions, and provide a baseline for reflection moving forward.

The second step in our predesign framework calls on providers and researchers to name the structural, environmental, and social barriers that may prevent the use of an mHealth intervention within a specific community and among specific users. This step is especially important in our contemporary moment when, following national and international attention to the disproportionate harms that police violence and poor access to quality health care have on the lives of Black and other minoritized people, providers and researchers are working to attune themselves to the health effects of structural racism; of course, this focus should always be a part of this research. In this step, we begin by considering questions of access to technologies, the affordability and sustainability of given devices, and whether or not they can be used safely and sustainably in a particular environment, and then we move onto situating these barriers within a structural context. We ask questions about the social forces that may shape individual behaviors, through both community engagement and feedback from the research team. The deliverable produced here should create a list of structural barriers to health that, in addition to reflections about the assumptions we are making about our technologies and patients’ capability to use them, can help shape the decisions we make next.

The third step in our predesign framework calls on providers and researchers to identify and pursue coalitions of stakeholders who can help enact the social, economic, and policy infrastructures necessary to sustainably deploy these mHealth interventions as designed. If our work in Step 2, for example, identifies how “digital redlining,” or the policies and investment decisions that “create and maintain class boundaries through strictures that discriminate against specific groups” [18], can impede patients’ use of an mHealth intervention, what kinds of research, policy expertise, and investment decisions might we need to address these issues [19]? Likewise, as we are working to identify relevant categories of expertise in this stage, how can we ensure that the expertise of our community partners informs these inquiries? And, finally, what concrete steps can we take to address these structural barriers to sustain our interventions? The deliverables produced here should include the formation of robust teams of experts as well as specific steps that can be taken to deploy this collective knowledge to address the social and policy environments that create the need for our innovative interventions and in which our patients and community partners live.

## Sociotechnical Tools to Address Environmental Barriers to Health

Our interview participants and community design team members in Flint, a predominantly Black and low-income city that is recovering from an economic downfall following the departure of the General Motors [20] automotive plant as well as an ongoing toxic water crisis [21,22], report that their capability and motivation to engage in physical activity to lower their blood pressure is often limited by their opportunity to do so. They identify 2 persistent barriers here: a dearth of safe outdoor spaces for exercise such as parks and sidewalks on which to traverse their neighborhoods, and the high cost of gyms and other indoor spaces where they might use exercise equipment for sustained physical activity. Even when park space was available, as one participant, a 61-year-old Black man told us, “I don’t see anything for seniors.” This absence of available public space made the challenge of affording a gym membership, even before the ongoing COVID-19 pandemic contributed to massive economic insecurity, more difficult. As another participant, a 52-year-old Black woman told us, “I would love to be able to have a gym membership. But there’s only a certain amount of income—I’m on a fixed income...But everything is so expensive, it’s difficult.”

Notably, neither a lack of access to spaces for physical activity nor limited financial resources presented as barriers to capability or opportunity for our participants in Ann Arbor, the affluent city in which the University of Michigan campus where we work is located. As another participant, a 48-year-old Asian woman, remarked, “There’s a lot of paths for walking, and we live near a playground so you can do something with the playground...They have equipment for you.” Likewise, income did not emerge as a barrier to physical activity within this population. Another participant, a 72-year-old White woman, said, “We’re fortunate we are retired, we have income, we aren’t dependent on the job anymore, we have a pension and social security. We have social security in the literal sense of secured money, and being able to afford a gym membership or any equipment.”

These are thorny issues involving complex interactions between race, class, income, geography, and public policy; if we seek to understand how technology can be used to promote healthy behaviors among patients, we must begin by acknowledging that these technologies are sociotechnical tools that, by definition, emerge from the interaction of these social forces. These technologies do not exist within a bubble and, as we argue here, neither can our efforts to design them.

## The Importance of Directly Addressing Structural Barriers to Health in mHealth Design

What we confront when we do health services research involving digital health tools is the fundamental tension between the promise of these devices and the ethos of innovation that spirits them, and the much more challenging realities of structural barriers to health that enable racial health disparities to persist. Melissa S. Creary [23] theorizes this tension through the concept of “bounded justice,” a phenomenon where the good intentions of justice-oriented stakeholders “are bounded by greater socio-historical constraints.” It is not enough, Creary writes, to pursue health equity and the amelioration of the indignities of longstanding health inequities simply through the distribution of “goods, materials, and resources” [23]. The political idealism of such interventions, even among so-called justice-based inclusive programs, comes with inherent limitations in its ability to repair “the underlying and deeper social inequalities embedded in individuals and communities, specifically those disadvantaged by racism,” when they fail to address “the underlying mechanisms that generated initial historical inequalities” in the first place [23]. We echo Creary’s call for more reflexive thinking about and action in the name of justice-oriented work that addresses these mechanisms as a part of our digital health technology intervention design.

Keeping these structural conditions at the forefront of our thinking about digital health design is vital because, lest we forget, we are not designing technical fixes to disease disparities but, rather, sociotechnical ones that must also engage with the social and policy environments that both necessitate innovation and constrain its deployment. This is why we argue early in this article that techno-pessimism and an effort to continue to improve digital health technologies constitute a compatible position and a requisite relationship with technology. The structural conditions that inhibit the sustained use of the tools we hope will help improve patient health should not dissuade us from seeking to improve care; rather, they should drive us to think more expansively, ethically, and systematically about this work. They should motivate us to foster collaborative relationships with policy experts, media and informatics scholars, and historians of medicine, as well as patients and caregivers with a wide range of interests in and objections to these kinds of technologies. They should center the role of structural racism in limiting access to lifesaving resources and in reproducing health disparities. And they should highlight opportunities for providers and researchers to contribute to the existing work that patient communities are engaging in to undo these structural harms.

Beginning from an acknowledgement that we are addressing “deep social problems” [24], which our technological interventions are simply unable to solve, enables us to identify social policy approaches that may help providers and patients to make the long-term improvements to health that they seek. This work must begin with an assessment of how we think about the role of technology in our research. As we are reminded by Amy Moran-Thomas’s [25] writing about the use of the pulse oximeter during the ongoing pandemic, we must be self-reflexive and critical of the tools we design and deploy to identify how our technologies might reproduce racial health disparities. And we must acknowledge that our focus on individual-level health behaviors, as Veinot et al [8] warn us, can only get us so far.

## Conclusions

Reframing how we think about our work—so that these issues and local contexts closely inform how we define our research problems, the kinds of solutions we pursue, and the changes we work to develop—can help us to bridge the divide between the techno-optimist and techno-pessimist positions in digital health research. We should be driven by this concern about “intervention-generated inequalities” to engage critically and productively with the promise of mHealth, ensuring that our work addresses the systems through which health disparities persist. We should think about the policy questions our data can illuminate, and make coalition building within and outside of our traditional networks of expertise an essential part of our work [2,26]. And when our work, by definition, involves the development and deployment of digital health technologies in an effort to improve health outcomes, we should integrate critical reflection about the technologies we deploy, the social contexts in which our patients and community partners live, and concerns about structural barriers to health as part of our efforts to design just and equitable health interventions.

## Abbreviations

CERB: Community Ethics Review Board
COM-B: Capability, Opportunity, Motivation, Behavior
mHealth: mobile health
WIRED-L: Wearables in Reducing Risk and Enhancing Daily Lifestyle
