# Correction: “Viewing Mobile Health Technology Design Through the Lens of Amplification Theory”

**DOI:** 10.2196/40273

**Published:** 2022-06-28

**Authors:** Beza Merid, Maria Cielito Robles, Brahmajee K Nallamothu, Mark W Newman, Lesli E Skolarus

**Affiliations:** 1 School for the Future of Innovation in Society Arizona State University Tempe, AZ United States; 2 Department of Neurology University of Michigan Medical School Ann Arbor, MI United States; 3 Department of Internal Medicine University of Michigan Medical School Ann Arbor, MI United States; 4 School of Information University of Michigan Ann Arbor, MI United States

In “Viewing Mobile Health Technology Design Through the Lens of Amplification Theory” (JMIR Mhealth Uhealth 2022;10(6):e31069) the authors have one correction.

In the original publication, the last name of author *'Maria Cielito Robles'* was listed as *'Cielito Robles'*. This has now been corrected to *'Robles.'*

This change is also reflected in the citation information of the paper as *'Robles MC.'*

The correction will appear in the online version of the paper on the JMIR Publications website on June 28, 2022, together with the publication of this correction notice. Because this was made after submission to PubMed, PubMed Central, and other full-text repositories, the corrected article has also been resubmitted to those repositories.

